# Lung cancer and tobacco smoking in Crete, Greece: reflections from a population-based cancer registry from 1992 to 2013

**DOI:** 10.1186/s12971-017-0114-2

**Published:** 2017-01-19

**Authors:** D. Sifaki-Pistolla, C. Lionis, V. Georgoulias, P. Kyriakidis, F. Koinis, S. Aggelaki, N. Tzanakis

**Affiliations:** 1 Clinic of Social and Family Medicine, School of Medicine, University of Crete, P.O. Box 2208, 71003, Heraklion, Crete Greece; 2 Department of Medical Oncology, School of Medicine, University of Crete, Heraklion, Crete Greece; 3 Department of Civil Engineering and Geomatics, Cyprus University of Technology, Limassol, Cyprus; 4 Department of Thoracic Medicine, School of Medicine, University of Crete, Heraklion, Crete Greece; 5 Cancer Registry of Crete, University of Crete, Heraklion, Greece

## Abstract

**Background:**

The Cancer Registry of Crete is a regional population database that collects cancer morbidity/mortality data along with several risk factors. The current study assessed the geographical variation of lung cancer among ever and never smokers in Crete during the last 20 years.

**Method:**

Lung cancer patient records (1992–2013) including information on medical history and smoking habits were obtained from the Cancer Registry of Crete. Age-Adjusted Incidence Rates (AAIR), prevalence of smoking among lung cancer patients and the Population-Attributable Fraction (PAF%) of tobacco smoking were estimated. Kaplan-Meier curves, grouped per smoking status were constructed, and spatio-temporal analyses were carried out to assess the geographical variations of lung cancer and smoking (a = 0.05).

**Results:**

New lung cancer cases in Crete accounted for 9% of all cancers (AAIR_both genders_ = 40.2/100,000/year, AAIR_males_ = 73.1/100,000/year, AAIR_females_ = 11.8/100,000/year). Ever smokers presented significantly higher incidence compared to ex-smokers (*p* = 0.02) and never smokers (*p* < 0.001). The highest increase was observed in ever smokers (AAIR_1992_ = 19.2/100,000/year, AAIR_2013_ = 25.4/100,000/year, *p* = 0.03), while never smokers presented the lowest increase from 1992 to 2013 (AAIR_1992_ = 5.3/100,000/year, AAIR_2013_ = 6.8/100,000/year, *p* = 0.2). The PAF% of lung cancer mortality is 86% for both genders (males: 89%, females: 78%). AAIRs ranged from 25 to 50/100,000/year, while significant geographical differences were observed among the municipalities of Crete (*p* = 0.02). Smokers living in the south-east urban regions presented higher risk of dying from lung cancer (RR = 2.2; 95%CI = 1.3–3.5).

**Conclusions:**

The constant increase of lung cancer rates among both genders, especially in females, outlines the need for targeted, geographically-oriented, life-style preventive measures. Design of population-based screening programs, tobacco awareness campaigns and smoking cessation programs in lung cancer hot spots could be guide by these findings.

## Background

Tobacco smoking is the leading preventable cause of morbidity and mortality worldwide [1]. Exposure to tobacco has a negative impact on health across life-course [1, 2]. Tobacco smoking is responsible for more than 5 million deaths per year, while it is estimated that this figure will increase to approximately 8 million deaths in 2030 [2]. Tobacco control has been addressed in many comprehensive cancer control strategies, since tobacco smoking accounts for 74% of trachea, bronchus, and lung cancers [3]. Lung cancer epidemic is now receding in several nations in which tobacco control has managed to reduce smoking, whereas it is rapidly rising in other regions [4].

Lung cancer incidence and mortality present geographic and temporal trends that are strongly determined by tobacco smoking pattern, which is considered the major risk factor in lung carcinogenesis [5]. Despite the success in identifying tobacco smoking as the major risk factor for lung cancer, this highly preventable disease remains among the most common and most lethal cancers globally, even in developed countries [4, 5]. Further studies on the relation of lung cancer with tobacco smoking are needed at a regional/country level to explore local particularities and understand the effects of risk factors [6–9].

Epidemiology of lung cancer in Greece has been extensively reported in the literature, with several studies suggesting that lung cancer has the highest disease-specific smoking Population Attributable Fraction (PAF%), ranging from 88.4 to 89% [10, 11]. However, community-based epidemiological studies are scarce in Greece. In addition, there are no national databases or studies that could have already undertaken these efforts. Systematic reports on cancer incidence and mortality are also considered a neglected issue. This gap is partially filled by the Cancer Registry of Crete (http://www.crc.uoc.gr), which is the only population-based regional cancer registry in Greece, with a big-database of more than two decades’ records [12]. The aim of registry is to monitor the progress of malignant neoplasms and report on cancer-attributed morbidity and mortality, identify cancer hot spots and estimate the future burden of the disease in Crete.

According to previous studies of the Cancer Registry of Crete, heterogeneous lung cancer trends are expected over Crete, regardless the island’s genetically homogeneous population [12]. Crete has been traditionally characterized by no racial or religious deviations, has mainly rural or semirural municipalities, as well as high occupational rates in the agricultural and business (i.e. tourism) sector. Furthermore, the nutritional habits of the Cretan population have been noted in the literature as being most beneficial for health [13]. The traditional Cretan diet was rich in vegetables, fruits, and olive oil [12, 13], but major changes have occurred during the last decades marking an increase in fast-food and sweets [14]. Contrary to their diet, Cretans usually show high rates of cigarettes and alcohol consumption among men, while the latest differences in lifestyle (increase of smoking for both genders, especially females; increase in occupational and environmental exposures along with the higher burden of multi-morbidity) are considered possible pathways through which individual characteristics might impact on lung cancer mortality [12, 15]. These variations should be assessed at a sub-regional level and further explored towards identifying potential clusters or significant differences of disease distribution in space.

The current study aimed to probe into the burden of lung cancer in Crete, according to the demographic characteristics and smoking habits (ever/never smokers) of lung cancer patients. Among its primary objectives was to monitor the geographical patterns of lung cancer incidence and mortality rates and assess smoking burden per municipality. Secondary objectives were to estimate the effect of smoking in Years of Potential Life Lost (YPLL) and attempt an in-depth exploration of the risk factors for lower survival rates.

## Methods

### Setting and data sources

The current population-based study was conducted in Crete, as a part of the Cancer Registry of Crete’s activities. The registry covers approximately 590,000 permanent residents, while administratively comprised of four counties and nineteen municipalities. The registry has recently enhanced its capacity and methodological approach of collecting and analyzing data. A new cancer monitoring system was developed towards this direction. Highly trained registrars are responsible for collecting data by distance, from two main sources of information: public hospitals for morbidity data, and death registries for mortality data. Data are directly coded according to the international classification system for Oncology (ICD10-O-2) [16]. Quality controls on the stored data are performed at three levels: a) automatically by the cancer monitoring system to identify and alert for duplicated or missing cases (in 100% of the data), b) by an epidemiologist to formulate the final version of the database with complete and qualitative records (follow-up in 100% of the data) and c) by an oncologist to randomly re-check 50% of the records per registrar back to the primary sources of information. An overall quantitative and qualitative control is performed annually towards measuring three major quality indicators that are widely recognized to enhance cancer control [17]: completeness = 98%, validity/accuracy = 97%, and timeliness = 99%. In addition, the capture-recapture method is used to capture potentially missing new cancer cases that haven’t attended a Cretan hospital. The main sources of information for this process are the Greek capital city hospitals and the Hellenic Statistical Authority. The identified missing percentage was 0.3% for lung cancer patients.

For the purposes of the current study, records on lung cancer from 1992 to 2013 were obtained from the cancer monitoring system. Data included information on patient’s demographic profile, personal and family medical history, and lifestyle factors (smoking habits, alcohol consumption). The main inclusion criteria were: 1) cases with primary lung cancer, 2) individuals that have been residing in Crete for at least the past 10 years, 3) a histologically or cytologically confirmed diagnosis of lung cancer.

### Measurements

Age-Standardized Incidence Rates (ASIR) and the Age-Standardized Mortality Rates (ASMR) were estimated using the direct standardization method, based on the European Standard population of 2011 [18]. All rates were estimated both for the entire island and per municipality of Crete, and were expressed as ASIR/100,000/year. In addition, prevalence (%) of smoking among lung cancer patients and smoking Population Attributed Fraction (PAF%) [19] of lung cancer mortality were estimated. Furthermore, the average smoking-attributable YPLL [20] for smokers and non-smokers was estimated per gender and age group.

### Statistical and spatial analysis

The analysis was performed in R (Design.9 module) and ArcMap 10.3.1., while all tests were conducted at a 0.05 level of significance. All rates and ratios were aggregated and mapped per municipality, while they were compared using the Kruskal-Wallis test (k > 2). A modified version of the local Moran’s Index [21–23] was computed to estimate the overall tendency of lung cancer data for spatial clustering according to smoking pattern status (ever smokers and never smokers). Moran’s Index is a statistic that quantifies spatial auto-correlation (similarity in space) of attribute values, and is used to estimate whether health parameters, such as lung cancer mortality data, are randomly distributed in space or exhibit tendency for (global) clustering. Moran’s index attains values from −1 to +1, indicating negative and positive spatial autocorrelation respectively. Furthermore, a zero value indicates a random spatial pattern with no overall clustering. The modified Index was proposed by Jackson et al., and is defined as [21]:

$$ {I}_W=\left(\frac{1}{Sy,{w}^2}\right)\frac{\underset{i}{\overset{N}{\varSigma }}\underset{\left\{j:\kern0.5em i\ne j\right\}}{\overset{N}{\varSigma }}wij\left(yi-\overline{y}\right)\left(yj-\overline{y}\right)}{\underset{i}{\overset{N}{\varSigma }}\underset{\left\{j:\kern0.5em i\ne j\right\}}{\overset{N}{\varSigma }}wij} $$

where yi are counts, y bar is the mean of the count data, and wij are interaction weights specifying neighborhood structure among polygons, defined as

$$ wij=\left(\begin{array}{cc}\hfill 1\hfill & \hfill \kern0.5em \mathrm{if}\kern0.5em i,j\kern0.5em \mathrm{are}\kern0.5em \mathrm{adjacent}\kern0.5em \mathrm{neighbors}\hfill \\ {}\hfill 0\hfill & \hfill \mathrm{otherwise}\hfill \end{array}\right. $$

and *Sy*, *w* ^2^ is an estimate of the data variance that depends on the weights *wij* as

$$ {s}_{y,w}^2=\frac{1\le i\overset{\varSigma }{<}j\le {N}^{wij{\left(yi-yj\right)}^2}}{\underset{i=1}{\overset{N}{\varSigma }}\underset{\left\{j:\kern0.5em i\ne j\right\}}{\overset{N}{\varSigma }}wij}. $$

In addition, 10-year Net-Survivals (%) were estimated for never and ever smokers per gender. Survival was measured from time of diagnosis until the date of death; multiple regression analysis was performed using Cox proportional hazards models. This procedure led to a survival prediction model by identifying the significant covariates to be included in the model (age: 25–44, 45–54, … < 85 years; gender: males, females; stage: I, II, III, IV, not known; personal medical history: no, lung disease, other cancer, not available/reliable; family medical history: no, lung cancer, lung disease, not available/reliable; smoking status: never smoker, ever smoker, not known). The survival prediction model was projected for 5 years and was internally validated by measuring both discrimination and calibration, using the concordance index (C-index) and a calibration curve respectively [24]. Both measures indicated the robustness of the prediction model (C-index = 0.86) [25].

## Results

### Burden of lung cancer in Crete

There were 5,509 primary lung cancer records in the Cancer Registry of Crete’s database from 1992 to 2013. Their characteristics are presented in Table 1. Most of them were males (87.3%) aged >65 years old (age group of 65–74; 35.2%) Additionally, most patients (45.5%) were diagnosed with stage IV and 16.1% with stage IIIA and IIIB. Approximately, 50.9% of the patients had previous medical diagnosis of other lung disease (mainly COPD) and 3.8% had a diagnosis of a second primary tumor. The family medical history of chronic lung diseases or lung cancer was also found to vary significantly among lung cancer patients (*p* = 0.04). Among those who had family medical history, 49.1% had a member of their family with COPD, or chronic bronchitis, or emphysema, while lung cancer was diagnosed in 18.5% of them.

**Table 1:** Characteristics of lung cancer patients in Crete (1992 to 2013)

Characteristics	Lung cancer cases		*P* value
	*N* = 5,509	%	
Gender			0.01
Males	4554	87.3	
Females	660	12.6	
Age group (at diagnosis)			<0.001
25–44	61	1.1	
45–54	263	4.8	
55–64	1135	20.6	
65–74	1935	35.2	
75–84	1754	31.9	
<85	357	6.4	
Stage (at diagnosis)			0.03
I	650	11.8	
II	457	8.3	
III	887	16.1	
IV	2507	45.5	
Not known	1008	18.3	
Personal medical history			0.04
No	2572	50.9	
Lung disease (COPD, Chronic Bronchitis, emphysema)	1728	34.2	
Other cancer (excluding lung cancer)	193	3.8	
Not available/reliable	564	11.2	
Family medical history			0.04
No	760	15	
LC	937	18.5	
Lung disease (COPD, Chronic Bronchitisemphysema)	2485	49.1	
Not available/reliable	875	17.3	
Smoking status*			0.01
Never smoker	925	16.8	
Ever smoker	4132	75.1	
Not known	452	8.1	
Alcohol consumption			0.04
No	1405	25.5	
Yes	1928	35.0	
Not known	2176	39.5	

*Pooled data: N = 5,057 lung cancer cases with information on smoking status

Furthermore, tobacco smoking was found to be significantly correlated with the lung cancer diagnosis (*p* = 0.01). The majority (75.1%) of lung cancer patients were ever smokers (current or ex smoker), while 16.8% were never smokers. Similar patterns were observed in lung cancer cases consuming alcohol, with 35% to be current or former consumers and 25% not to consume alcohol (*p* = 0.04).

**Fig. 1:**
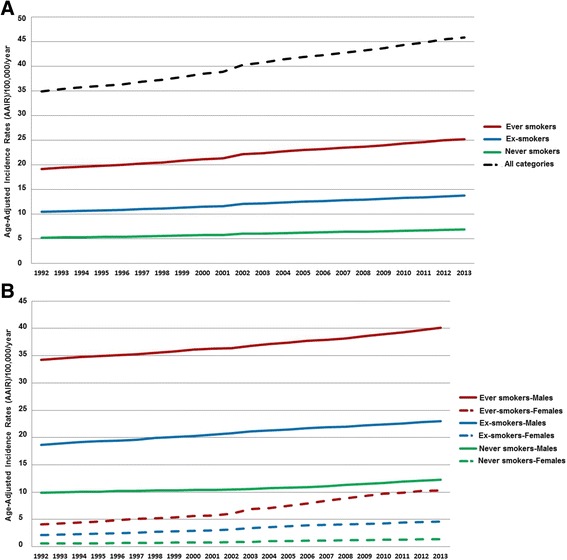
Age-Adjusted Incidence Rates per smoking category (**a**) and gender (**b**) in Crete from 1992 to 2013

The AAIR varied significantly from 1992 to 2013, for both genders (AAIR_1992_ = 34.9/100,000, AAIR_2013_ = 45.9/100,000, *p* = 0.02). In addition, lung cancer was more frequent (*p* = 0.01) in males (AAIR = 73.1/100,000/year) compared to females (AAIR = 11.8/100,000/year). Nevertheless, females presented higher proportional increasing trends (AAIR_1992_ = 8.2/100,000, AAIR_2013_ = 18.9/100,000, *p* < 0.001), especially after 2008 compared to males. Major differences were observed according to smoking status, as illustrated in Fig. 1a. Ever smokers presented significantly higher incidence of lung cancer compared to ex-smokers (*p* = 0.02) and never smokers (*p* < 0.001). All rates presented increasing trends with a significant peak in 2001–2002 (*p* = 0.03). The highest increase of approximately 6 new cases/100,000 population was observed in ever smokers (AAIR_1992_ = 19.2/100,000/year, AAIR_2013_ = 25.4/100,000/year, *p* = 0.03), while never smokers did not present a significantly different increase of lung cancer incidence from 1992 to 2013 (AAIR_1992_ = 5.3/100,000/year, AAIR_2013_ = 6.8/100,000/year, *p* = 0.2) (Fig. 1b). Male ever smokers had greater AAIR (AAIR_1992–2013_ = 36.8/100,000/year), while male never smokers (AAIR_1992–2013_ = 10.7/100,000/year) presented slightly higher rates compared to female ever smokers (AAIR_1992–2013_ = 6.9/100,000/year). Notably, there was no significant difference of lung cancer incidence in female ex-smokers and never smokers during the period 1992–2013, contrary to the statistically significant increase of the incidence of lung cancer in ever smokers (AAIR_1992_ = 4.1/100,000/year, AAIR_2013_ = 10.3/100,000/year, *p* = 0.03).

### Geographical variation of lung cancer and smoking habits

**Fig. 2:**
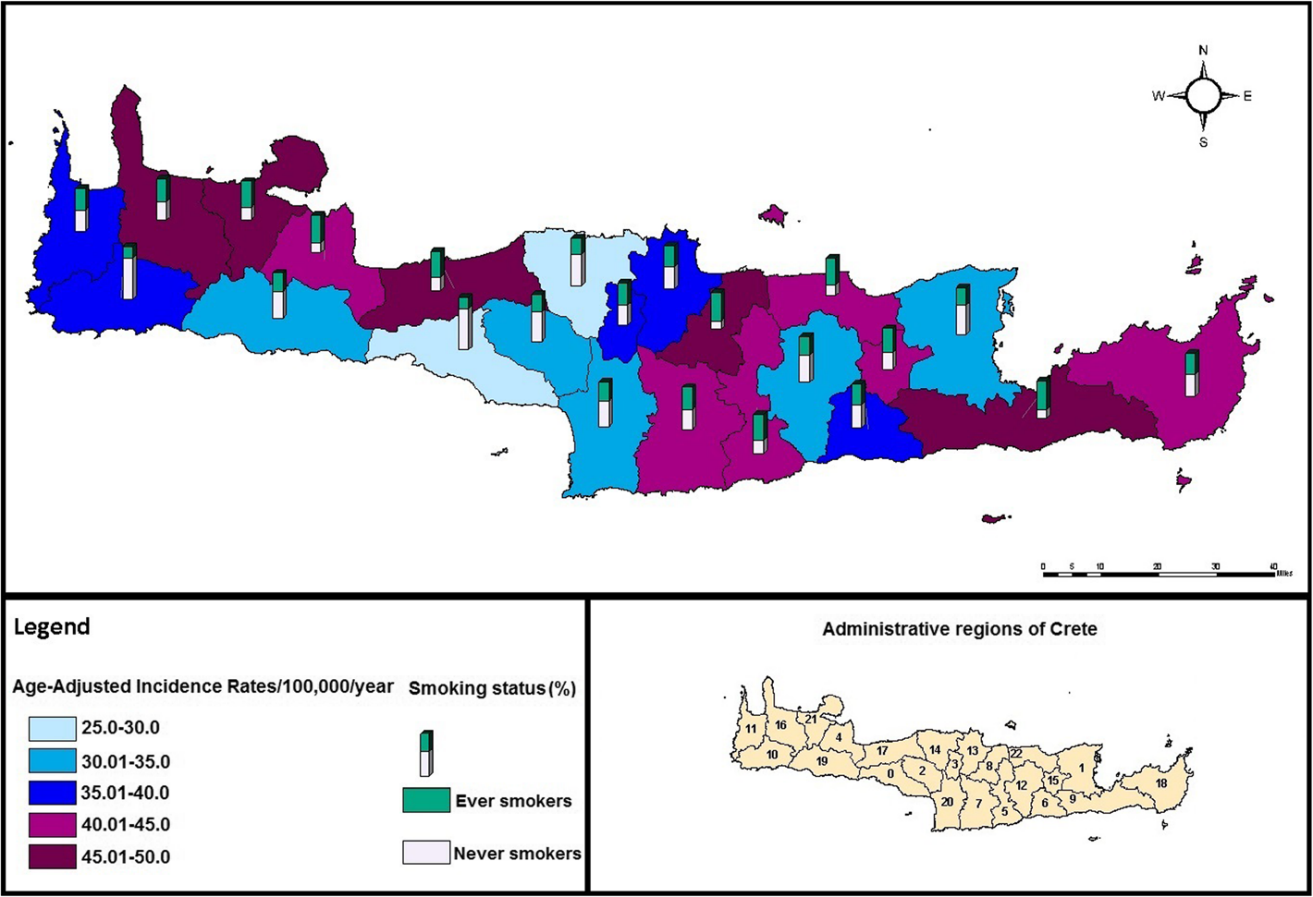
Geographical distribution of Age-Adjusted Incidence Rates/100,000/year and smoking proportion in Crete

The spatial variation of AAIRs is presented in Fig. 2. Rates ranged from 25/100,000/year to 50/100,000/year, while significant geographical differences were observed among the municipalities of Crete (*p* = 0.02). Municipalities with codes 8; 9; 16; 17 and 21 presented the highest lung cancer burden. In addition, the municipalities of south-east Crete were found to have increased incidence of lung cancer (AAIRs = 40.01-45/100,000/year). Furthermore, two municipalities (codes: 0; 13) presented the lowest lung cancer incidence, ranging from 25 to 30 new cases/100,000/year. Overall, municipalities with medium or high incidence rates (40.01-50/100,000/year) presented higher percentage of ever smokers (≥55%, *p* < 0.001).

**Fig. 3:**
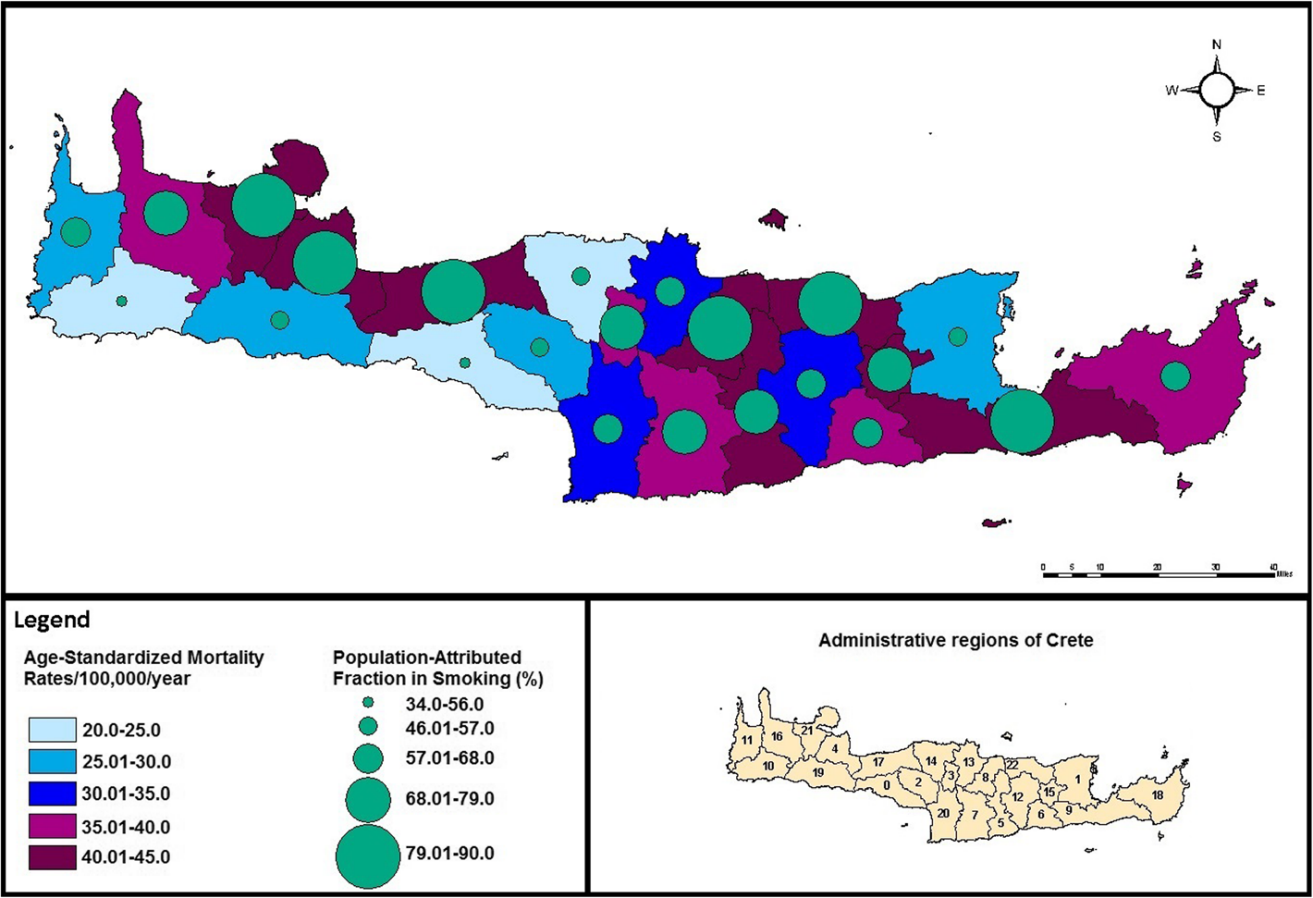
Geographical distribution of Age-Standardized Mortality Rates/100,000/year and PAFs (%) in Crete

ASMRs along with the PAFs are mapped per municipality in Fig. 3. ASMRs vary significantly among municipalities (ASMRs = 20-45/100,000/year, *p* = 0.01). Eight municipalities (codes: 4; 5; 8; 9; 15; 17; 21 and 22) were found to have increased mortality (ASMRs = 40.01-45/100,000/year) compared to the lowest rates observed in the south-west municipalities of the island, as well as in municipalities encoded 1 and 14 (ASMRs = 20-30/100,000/year). The PAFs (34-90%) seem to follow the lung cancer mortality pattern, with municipalities of increased burden presenting significantly higher PAFs (*p* < 0.001). In municipalities encoded 8 and 9 PAFs reach 87.9% and 86.3%, respectively.

When exploring overall lung cancer clustering according to smoking patterns, results from the Moran’s Index yielded a high power of 98% with potential clusters including 30% of the population and an overall positive value of spatial autocorrelation (Morans’I = 0.7). The central and south-east municipalities of the island presented a higher risk for lung cancer (RR = 2.2. 95% CI = 1.3-3.5).

### Impact of smoking to lung cancer YPLL

Furthermore, Table 2 demonstrates the average YPLL and PAFs attributed to lung cancer, according to gender and age in Crete. Males had higher PAF (89%), compared to females (78%). Accordingly, male ever smokers presented slightly higher YPLL (16) than female (YPLL = 15). Nevertheless, females (YPLL_never smokers_ = 9; YPLL_ever smokers_ = 15) compared to males (YPLL_never smokers_ = 11; YPLL_ever smokers_ = 16), presented a higher difference of YPLLs between never and ever smokers. In addition, ever smokers presented significantly higher YPLL (*p* = 0.03), regardless of age. The age groups of 25–39 years and 40–54 years were found to have increased YPLL for both smoking categories, while lung cancer patients aged 40–54 years and 55–64 years had the highest difference of YPLL between never and ever smokers (40–54 years: YPLL_never smokers_ = 15; YPLL_ever smokers_ = 23; 55–64 years: YPLL_never smokers_ = 7; YPLL_ever smokers_ = 15). Furthermore, PAFs ranged from 57 to 92 according to age group, with elder individuals to present higher portions (91-92% among >65 years).

**Table 2:** Average YPLL and PAFs (%) between genders and among age groups*

Characteristics	Average YPLL (years)		PAF (%)
	Never smokers	Ever smokers	
Gender			
Males	11	16	89
Females	9	15	78
Age group (at year of diagnosis)			
25–39	35	40	57
40–54	15	23	88
55–64	7	15	89
65–79	4	7	91
> 80	0	0	92

*Pooled data: N = 5,057 lung cancer cases with information on smoking status

### Survival and lung cancer risk factors

Table 3 presents the results of the Cox multivariate regression analysis in lung cancer patients, which identified significant covariates for inclusion in the prediction survival model. Males (β = 0.6; 95%CI = 1.2-1.4) and patients at stage IV (β = 1.6; 95%CI = 4.2-4.4) were more likely to die from lung cancer. Higher age (β = 0.9; 95%CI = 1.9-2.3), personal and family medical history (β = 0.9; 95%CI = 1.3-2.3 and β = 0.5; 95%CI = 1.1-1.5 respectively) and ever smokers (β = 1.8; 95%CI = 1.3-1.7) presented significantly higher risk.

**Table 3:** Cox Proportional Hazards Multivariate Regression Analysis in lung cancer patients in Crete

Covariates	β coefficient	Hazard ratio	95% CI	*P* value
Males	0.6	1.3	1.2–1.4	0.01
Age	0.9	2.1	1.9–2.3	0.01
Stage II	0.7	1.4	1.1–1.7	0.03
Stage III	0.7	1.4	1.2–1.6	0.02
Stage IV	1.6	1.9	1.8–2.0	<0.001
Personal medical history	0.9	1.8	1.3–2.3	0.03
Family medical history	0.5	1.3	1.1–1.5	0.02
Ever smoker	1.8	1.5	1.3–1.7	0.01
Alcohol consumption	0.3	1.1	0.4–1.8	0.8

**Fig. 4:**
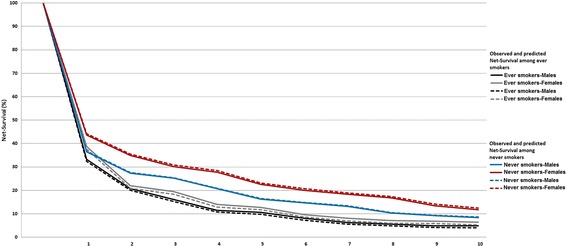
Observed and predicted lung cancer Net-survival among never and ever smokers, in Crete

Figure 4 illustrates the Net-Survival (%) of lung cancer patients in Crete, after the first 10 years from diagnosis. Overall, females had better survival rates compared to males, both in ever (5 years Net-survival_females_ = 12.8%; 5 years Net-survival_males_ = 10.6%) and never smokers (5 years Net-survival_females_ = 22.5%; 5 years Net-survival_males_ = 16.3%). Similar differences were expected within the next 7 years according to the predicted Net-survival (ever smokers: 5 years Net-survival_females_ = 11.7%; 5 years Net-survival_males_ = 9.8% and never smokers: 5 years Net-survival_females_ = 23.1%; 5 years Net-survival_males_ = 16.5%). Nevertheless, predicted survival indicated slight decreases for both genders compared to the observed one, especially in female ever smokers even from the first year after diagnosis (Observed survival: 1 year Net-survival_females_ = 38.7%; 1 year Net-survival_males_ = 33.2% and predicted survival: 1 year Net-survival_females_ = 37.6%; 1 year Net-survival_males_ = 32.4%).

## Discussion

### Key findings

This population-based epidemiological study provided new evidence on lung cancer and tobacco smoking in Crete. The major findings of this study conveyed new evidence on lung cancer burden and its geographical variation in Crete, while they reflected the strong impact of smoking on lung cancer survival. In particular, the major findings of this study were: a) the heterogeneous geographical pattern of lung cancer, with municipalities of central and south-east Crete that presented significantly higher rates especially for ever smokers; b) the observed temporal increases of lung cancer, more significantly among males, and the rapidly increasing rates among female ever smokers after 2001; c) the great number of YPLL in males, as well as the significant difference of YPLLs among female ever and never smokers; d) the significant impact of tobacco smoking on lung cancer incidence and patients’ survival.

### Discussion in the context of the literature

Tobacco smoking is widely recognized as the main cause of premature death from lung cancer worldwide [26–28]. The causal relationship of lung cancer and smoking was established in the 1950’s [26]. Since then, a large number of studies in the literature have revealed the impact of tobacco smoking on lung cancer [27–31]. In particular, the highest risk for lung cancer mortality in ever smokers has been found to be approximately 9 times higher than in never smokers [32]; much higher than the identified risk in the current study. Similarly to our study, Tammemagi et al., have stressed that smoking is an important independent predictor of short-term lung cancer survival [27]. Furthermore, age was found to be a significant risk factor in the current study. Larger proportions of lung cancer incidence were observed among patients of age 55 and over. This finding is in accordance with the literature especially among ever smokers of this age group [32, 33].

In addition, place of residence seems to be a core component of lung cancer incidence and mortality trends, as highlighted by the current and other studies [34, 35]. It is obvious that a wide range of factors, such as demographic, occupational and environmental, are linked with rurality. These factors may have hidden effects on lung cancer epidemics; such effects are more severe in geographical regions where prevalence of smoking is higher (e.g., urban or semi-urban municipalities) [35]. Geographical inequalities in lung cancer incidence distribution may be observed due to the different smoking habits between rural and urban regions. Marked differences in tobacco smoking patterns have been found in Greece, among different geographical regions (rural vs urban) and within age groups and genders [36]. Furthermore, the vast majority of Cretans consider smoking a part of their culture which is strongly linked with social activities of men, especially while socializing in traditional “kafeneia” or modern cafes during the day [37]. This habit increases both active and passive smoking in public and private working spaces, as well as inside the house, especially in urban, densely populated regions. Moreover, even though most of the municipalities in Crete are characterized as rural or semi-urban, their residents still have a diverse demographic profile; this leads to inequalities even between rural regions of the same county [15]. Smoking prevalence in these regions may have affected the lung cancer incidence rates, since, as mentioned in the literature, individuals over 70 present significantly lower smoking rates, or die from other causes due to the lag period between tobacco exposure and disease onset [38]. Apart from smoking, other occupational and environmental risk factors may have an additional effect on the increased lung cancer burden in certain rural municipalities that presented high lung cancer incidence and mortality. Greenhouse intense farming is among the most common agricultural practices in some rural and semi-urban municipalities (codes: 8; 9; 22). Studies has already shown the increased risk of lung cancer mortality and lower survival among individuals exposed to pesticides at work, especially among female greenhouse workers [39, 40]. Previous studies have documented the association of pesticides/greenhouse farming and the development of chronic disease [41]. The projected lower survival rates in the current study raise concern towards this direction. Nevertheless, further research and additional field studies on the joint effect of smoking and other risk factors (e.g. environmental exposures, lifestyle habits etc.) are required in order to clearly demonstrate the existence of such association in Crete.

YPLL due to lung cancer are higher in male ever-smokers in most studies, while they present constantly increasing temporal trends (1952: 6.6; 2012: 11.3) [42]. Nevertheless, the proportion of increase during the last decades is higher in Cretan and Greek female ever-smokers, indicating an urgent need for comprehensive smoking policies that focus on women [43]. Public health policies for tobacco control should consider the time lag in the tobacco epidemic among women compared to men, the differences in lifestyle and the local particularities and needs per geographical region [43].

### Impact of this study

The increasing rates of tobacco smoking and lung cancer in Crete along with the projected lower survival rates have stressed the need for integrated and effective cancer control with emphasis on screening [42]. Establishment and acceptance of lung cancer screening as a public health policy is still a neglected issue in Crete. This is either due to low health literacy of the general population, or due to accessibility issues. Other concerns include risks of radiation, over diagnosis bias, proportion of false positives and cost benefit analysis. Additionally, barriers in successful engagement to smoking cessation programs may have negatively affected lung cancer trends in Crete. The findings of this study will be utilized by the Region of Crete and the Health Region of Crete, to boost smoking cessation [42, 43] in the island and arm mobile lung cancer screening units in high risk municipalities.

Furthermore, the Cancer Registry of Crete aims to develop a multidisciplinary team of health care professionals to intervene in population groups at risk, and conduct filed research to identify additional lung cancer risk factors. General Practitioners (GPs) will have a key role in this team with the aim to monitor lung cancer patients, provide integrated and continuous care or rehabilitation services, and raise awareness in the catchment area of their Primary Health Care (PHC) unit and promote healthy living in smoke-free places [44].

### Strengths and limitations

Although this is the first study in Crete incorporating data for more than 20 years, and the only one in Greece on lung cancer incidence and tobacco smoking, a number of limitations may exist. Among these, the lack of data on other potential risk factors (e.g. behavioral, occupational, and environmental) may have led to an over-estimation of the impact of tobacco smoking on lung cancer incidence despite the fact that our data are in agreement with those of the literature [27, 32]. All rates were aggregated at the municipality level, therefore the exploration of causal relations between the exact place of residence and lung cancer incidence is limited.

Nevertheless, the geographical distribution of lung cancer rates and PAFs per municipality revealed numerous spatial inequalities that provide valuable insights to healthcare professionals, researchers and authorities. Furthermore, this study differs from most of the studies on lung cancer and tobacco smoking, since its findings were exported from primary individual data. Last, it should be noted that, as the Cancer Registry of Crete’s database has undergone several quality controls, it constitutes a reliable data base, and hence offers a robust basis for studying the association of smoking with lung cancer in the island of Crete. The reliable database of the registry has undergone several quality controls as mentioned in the methods section. Therefore, it is a robust tool for assessing the association of smoking with lung cancer.

## Conclusions

The findings of the current study provided rigorous evidence that supports the strong relation between lung cancer and tobacco smoking in Crete. The constantly increasing trends of lung cancer incidences among ever smokers, the identification of high risk areas and the projected survival trends provided new evidence on the current knowledge of lung cancer epidemiology in Crete. Evidence conveyed from this study could support the design of targeted population-based screening programs, lead tobacco awareness campaigns and plan smoking cessation programs in lung cancer hot spots. Future research will enhance knowledge on lung cancer epidemics by exploring other risk factors such as environmental, socio-economic and behavioral ones.

## Abbreviations

AAIR: Age-adjusted incidence rates
ASMR: Age-standardized mortality rates
ICD: International classification of disease
PAF: Population-attributable fraction
YPLL: Years of potential life lost
